# Performance and Clinical Utility of Deep Learning for Detecting Referable Age-Related Macular Degeneration on Fundus Photographs: A Systematic Review and Meta-Analysis

**DOI:** 10.3390/diagnostics16040633

**Published:** 2026-02-22

**Authors:** Wei-Ting Luo, Ting-Wei Wang

**Affiliations:** 1 Department of Ophthalmology, Kaohsiung Veterans General Hospital, Kaohsiung 813414, Taiwan; 2 Department of Medical Education, Taipei Veterans General Hospital, Taipei 112304, Taiwan; 3 School of Medicine, National Yang-Ming Chiao Tung University, Taipei 112304, Taiwan; 4 Institute of Biophotonics, National Yang-Ming Chiao Tung University, Taipei 112304, Taiwan; 5 Department of Computer Science, Whiting School of Engineering, Johns Hopkins University, Baltimore, MD 21218, USA

**Keywords:** age-related macular degeneration, deep learning, fundus photography, diagnostic accuracy, screening, meta-analysis

## Abstract

**Background/Objectives:** Age-related macular degeneration (AMD) is a leading cause of irreversible central vision loss in older adults. Detection of referable AMD—typically intermediate or advanced disease requiring specialist evaluation—is critical for timely intervention. Deep learning (DL) applied to color fundus photographs has emerged as a potential tool to support large-scale AMD screening. This systematic review and meta-analysis evaluated the diagnostic accuracy of DL algorithms for detecting referable AMD and compared their performance with human graders. **Methods**: We systematically searched PubMed, Embase, Web of Science, and IEEE Xplore through 18 December 2025. Diagnostic accuracy studies assessing DL algorithms on color fundus photographs for referable AMD in adults were included. Two reviewers independently screened studies, extracted data, and assessed risk of bias using an AI-adapted PROBAST framework. Pooled sensitivity and specificity were estimated using a bivariate random-effects model. Clinical utility was evaluated using likelihood ratios, and paired head-to-head comparisons were synthesized using a contrast-based meta-analysis. **Results:** Fourteen studies were included. DL algorithms achieved a pooled sensitivity of 0.91 (95% CI: 0.86–0.94) and specificity of 0.93 (95% CI: 0.86–0.96), with substantial heterogeneity. The pooled positive and negative likelihood ratios were 12.22 and 0.10, respectively, indicating strong diagnostic utility. In direct comparisons, DL systems showed slightly lower sensitivity but higher specificity than human graders. **Conclusions:** Deep learning demonstrates high diagnostic accuracy for detecting referable AMD from fundus photographs and may support screening and referral workflows. Further prospective validation and standardized evaluation are needed before widespread clinical implementation.

## 1. Introduction

Age-related macular degeneration (AMD) is a leading cause of irreversible central vision loss among older adults worldwide and is expected to impose an increasing public health burden as populations age [1]. The number of individuals affected by AMD is projected to rise substantially over the coming decades, underscoring the importance of scalable strategies for early detection and timely referral [1]. Clinically, “referable” AMD generally denotes intermediate or advanced disease that warrants specialist evaluation and/or closer monitoring, because these stages carry a substantially higher risk of progression to late AMD and vision-threatening complications such as neovascular AMD and geographic atrophy [2,3]. Early identification of referable AMD may enable timely intervention—particularly rapid initiation of anti-vascular endothelial growth factor (anti-VEGF) therapy for neovascular AMD and risk-modifying strategies for intermediate disease—thereby reducing preventable vision loss and associated disability [2,3,4].

Despite these needs, systematic screening for referable AMD at the population scale remains challenging. Retinal imaging volumes have expanded rapidly in both community and health-system settings, yet expert grading capacity is limited and variability across readers and clinical environments is non-trivial [5,6]. Fundus photography remains widely available and cost-effective, while optical coherence tomography (OCT) provides additional structural information that improves sensitivity for exudative changes and supports clinical decision-making [7,8]. However, both modalities still depend on trained human interpretation, creating bottlenecks for broad deployment of AMD screening programs—especially in aging societies and regions with uneven access to retina specialists [5,9].

Deep learning (DL), particularly convolutional neural networks (CNNs), has emerged as a powerful approach for automated analysis of retinal images, learning discriminative representations directly from pixel-level data without manual feature engineering [10,11]. Over the last decade, DL systems have demonstrated high diagnostic performance for multiple retinal diseases and have progressed from proof-of-concept experiments to clinically oriented systems evaluated in large datasets [6,10,12]. For AMD, early seminal work showed that CNN-based models trained on large fundus datasets could identify referable AMD with high AUC and accuracy, approaching performance of trained graders [13,14]. Subsequent studies have expanded AMD detection across diverse imaging devices and populations, including models trained on standardized datasets such as AREDS and models adapted for real-world or lower-cost imaging platforms [14,15,16].

In parallel, several investigations have conducted direct comparisons between DL algorithms and human experts, an essential step for understanding clinical readiness and workflow integration. Prior reports indicate that DL-based AMD classifiers can achieve performance comparable to ophthalmologists and retinal specialists, and in some settings may offer improved consistency or sensitivity—although results can vary depending on case mix, reference standards, and disease definitions [6,13,17]. Beyond diagnostic classification, DL has also been used to predict future conversion to neovascular AMD, highlighting the broader potential of AI beyond cross-sectional detection [18]. Nevertheless, the literature remains heterogeneous, with differences in study design, validation strategy, thresholds for “referable” AMD, imaging modality, and the involvement of vendors or proprietary datasets—all of which can influence reported accuracy and generalizability [11,19,20].

Accordingly, we conducted a systematic review and meta-analysis to quantify the diagnostic accuracy of DL for detecting referable AMD. By consolidating recent evidence, this work aims to clarify the pooled diagnostic performance of DL systems for referable AMD and contextualize their effectiveness relative to human expertise, thereby informing future implementation and evaluation in real-world screening and referral pathways [11,17,20].

## 2. Materials and Methods

### 2.1. General Guideline

We conducted this systematic review and meta-analysis to evaluate the diagnostic accuracy of deep learning algorithms for detecting referable AMD from color fundus photographs, adhering strictly to the Preferred Reporting Items for Systematic Reviews and Meta-Analyses of Diagnostic Test Accuracy (PRISMA-DTA) guidelines [21]. The study protocol was registered with INPLASY (registration number: INPLASY2025120099) [22], and the PRISMA checklist is provided in Supplemental Tables S1 and S2. Institutional Review Board (IRB) approval and informed consent were not required, as this study utilized only aggregate data from previously published literature.

### 2.2. Database Searches and Identification of Eligible Manuscripts

A systematic literature search was independently conducted by two reviewers (L-WT, T-WW) to identify studies comparing the performance of deep learning algorithms against expert clinical assessment for the diagnosis of referable age-related macular degeneration (AMD) using color fundus photography. Electronic searches were performed across four major databases—PubMed, Embase, Web of Science, and IEEE Xplore—from their inception through 18 December 2025, without language restrictions.

The search strategy employed terms related to artificial intelligence (e.g., ‘Deep Learning,’ ‘Convolutional Neural Network,’ ‘Transformer,’ ‘ViT’), AMD (e.g., ‘Macular Degeneration,’ ‘Geographic Atrophy,’ ‘Drusen’), and imaging modalities (e.g., ‘Fundus Photography’). The full search strategy is detailed in Supplementary Table S3.

Study screening was managed in EndNote. After duplicate removal, the deduplicated library was used for title/abstract screening and full-text review, which were performed independently by two reviewers (L-WT, T-WW); disagreements were resolved by consensus. We included diagnostic accuracy studies evaluating deep learning algorithms for detecting referable AMD using color fundus photographs (CFP) as the sole imaging input in adult or real-world screening populations. Eligible CFP inputs included conventional desktop CFP, portable/smartphone-based CFP, and ultrawide-field color fundus images when reported. Studies were excluded if the index test used additional ophthalmic imaging modalities (e.g., OCT or fluorescein angiography) or multimodal imaging inputs (e.g., CFP + OCT); for studies described as “multimodal” AI (e.g., vision-language MLLMs), eligibility required that the only imaging input remained CFP. We also excluded studies focused exclusively on pediatric populations, non-deep learning methods, non-CFP imaging, as well as review articles, conference proceedings/abstracts, brief reports, and retracted publications.

### 2.3. Data Extraction and Management

Two reviewers (T-WW, L-WT) independently extracted data from eligible full texts using a standardized form. The extracted data were organized into several comprehensive domains: study characteristics (including first author, year of publication, country of origin, study design, temporal period, and healthcare setting), patient demographics (comprising mean age, gender distribution, total sample size, number of images, and economic status), and the precise definition of the target condition (referable AMD). In terms of technical specifications, we documented detailed information regarding the imaging equipment—such as the type of fundus camera (desktop, smartphone-based, or ultrawide field), field of view (e.g., 30°, 45°, or 200°), as well as the characteristics of the deep learning systems, including the specific architecture (e.g., ResNet, Inception, or Transformers), software vendor involvement, and training dataset size. Additionally, we extracted critical outcome data, including the reference standard employed for ground truth (such as central reading center adjudication versus clinical consensus) and diagnostic performance metrics (sensitivity, specificity, and raw confusion matrix values). Any discrepancies encountered during this process were resolved through discussion to reach a consensus or by arbitration with a third reviewer.

### 2.4. Quality Assessment

Risk of bias was assessed independently by two reviewers (T-WW, L-WT) using the Prediction model Risk Of Bias Assessment Tool (PROBAST) [23], applied within an AI-specific framework (PROBAST-AI). We evaluated four domains: (1) Participants (data source representativeness, eligibility criteria, and selection methods); (2) Predictors (definition of model inputs, timing, and consistency with intended use); (3) Outcome (reference standard definition, grading procedures, and blinding); and (4) Analysis (sample size adequacy, handling of overfitting/data leakage, internal vs. external validation, and appropriateness of statistical analysis, including clustering by eye when applicable).

Each domain was rated as Low, High, or Unclear risk of bias. Unclear risk was assigned when reporting was insufficient to judge the domain as Low or High (e.g., incomplete description of sampling and exclusions, missing details on adjudication/blinding, lack of transparency on prespecified thresholds, unclear handling of ungradable images, or unreported methods for accounting for within-patient correlation when both eyes or multiple images per patient were analyzed). The overall risk of bias for each study was determined by the highest risk rating across domains (i.e., any High domain→overall High; otherwise, any Unclear domain→overall Unclear; otherwise, overall Low).

We also prespecified AI-relevant methodological concerns, including inappropriate inclusion of bilateral eyes without statistical adjustment, small test sets relative to model complexity, lack of external validation, and implausibly high performance suggestive of overfitting or data leakage.

### 2.5. Statistical Analysis

We constructed 2 × 2 contingency tables for each study to extract the counts of true positives, false positives, true negatives, and false negatives. Pooled estimates of sensitivity and specificity, with 95% confidence intervals (CIs), were calculated using a bivariate random-effects logistic regression model. This approach accounts for the correlation between sensitivity and specificity across studies to evaluate threshold effects. Diagnostic performance was visualized using forest plots and Summary Receiver Operating Characteristic (SROC) curves. To investigate potential sources of heterogeneity, subgroup analyses were conducted based on the following covariates: AI software architecture, economic status (World Bank classification) [24], healthcare setting (primary vs. tertiary), dataset (public dataset vs. private dataset), validation method (internal vs. external), unit of analysis (patient vs. eye), camera type (desktop vs. smartphone), presence of other eye disease targets, reference standard criteria, disagreement resolution method, software certification status, vendor involvement, region of testing, ungradable image handling, quality of study and publication type.

Furthermore, we performed a contrast-based meta-analysis to benchmark the diagnostic performance of AI algorithms directly against human graders. For studies providing head-to-head comparisons, we extracted paired 2 × 2 contingency data—indexing AI performance alongside human performance within the same cohort—to calculate relative performance metrics. This contrast-based approach was chosen to mitigate the bias associated with indirect comparisons. Publication bias was assessed using Deeks’ funnel plot asymmetry tests [25]. To gauge clinical applicability, we constructed likelihood ratio scattergrams and Fagan nomograms. These tools illustrated the relationship between likelihood ratios and the modification of pre-test probabilities for referable AMD following diagnostic outcomes. All statistical analyses were conducted using Stata version 18.0 (StataCorp, College Station, TX, USA), employing the metadta [26] and midas packages

## 3. Results

### 3.1. Study Identification and Selection

**Figure 1:**
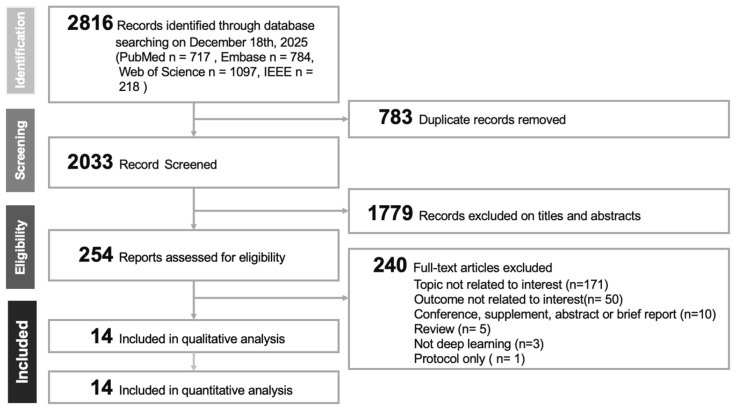
PRISMA flowchart of included studies.

Our systematic literature review, depicted in the PRISMA flowchart (Figure 1), began with a search across PubMed, EMBASE, and web of science, IEEE, yielding 2816 studies. After removing 783 duplicates—defined as records referring to the same citation/study retrieved from multiple databases (identified via matching title/author/year and/or DOI/PMID and confirmed when necessary)—we screened 2033 articles using EndNote, excluding 1779 for insufficient relevance. Further scrutiny of 240 full-text articles led to additional exclusions for various reasons. Ultimately, this process resulted in selecting 14 studies [2,4,5,6,7,10,11,27,28,29,30,31,32] for our meta-analysis.

### 3.2. Overview of Study Characteristics

Table 1 summarizes the basic characteristics of the 14 studies included in this review evaluating artificial intelligence-based screening for referable age-related macular degeneration (AMD). These studies were conducted across diverse geographical regions, predominantly in high-income countries such as the United States [3,5,10,27,32], Australia [29], Taiwan [2], France [31], Germany [7], and Singapore [6]. Studies involving middle- and low-income contexts were conducted in India [11,28] and China [4]. The majority employed retrospective cohort designs [2,3,5,6,7,10,11,27,29,30,31,32], while two studies utilized prospective validation cohorts [4,28]. Most study populations comprised adults and older adults typical of AMD screening/clinical contexts; where reported, mean/median ages generally fell in the sixth to eighth decades, although one large-scale health-check cohort included a broader age range (median 42; range 8–87) [4].

Female participation rates varied from 44.3% to 69.7% [4,6,11,27,28,29,31], although gender distribution was not reported in several datasets utilizing public repositories. Sample sizes varied significantly, ranging from small clinical cohorts of fewer than 100 patients [27,29] to large-scale screening datasets exceeding 100,000 images or participants [4,5,7]. Healthcare settings were diverse, spanning primary care clinics [4,6,29], tertiary referral centers [2,27,28,31,32], and mixed settings utilizing public datasets [7,11,30]. While all studies assessed referable AMD, several also evaluated concurrent ocular conditions, including diabetic retinopathy and glaucoma [4,6,29,30]. Study-specific definitions of referable AMD, handling of ungradable images, and analytic units varied and are summarized in Supplementary Table S4.

### 3.3. Comparative Overview of Deep Learning Algorithms in Diagnostic Studies

Across all 14 studies (Supplementary Table S5), the reference standard for ground truth was established through human expert grading, typically relying on AREDS classification schemes (e.g., simplified, 4-step, or 9-step scales) [2,3,5,7,10,11,27,28,31,32]. Disagreements in grading were predominantly resolved through adjudication by senior retinal specialists or consensus panels. Image acquisition was performed primarily using desktop fundus cameras (e.g., Canon, Topcon, Zeiss) with a standard 30° to 45° field of view [2,4,5,6,7,10,29,30,31,32]. However, recent studies have expanded into novel modalities, including smartphone-based imaging (Remidio Fundus on Phone) utilized in two studies [11,28] and ultrawide-field (UWF) imaging (200° Optos) employed by Most et al. [27]. Regarding image quality control, the majority of studies excluded ungradable images from the final analysis, with the exception of Ting et al. [6], who classified ungradable images as referable, and Grassmann et al. [3], who treated them as a distinct class. The deep learning architectures were overwhelmingly based on Convolutional Neural Networks (CNNs), utilizing backbones such as ResNet [10], Inception-V3 [31,32], VGGNet [6], DenseNet [2], and EfficientNet [11,28]. A notable departure from standard CNNs was observed in Most et al. [27], which evaluated a vision-language multimodal large language model (MLLM) on ultrawide-field color fundus images. Several studies assessed commercially available or certified algorithms, including RetCAD [29,30], VeriSee™ AMD [2], Medios AI-AMD [28], and SELENA+ [6].

### 3.4. Quality Assessment

Risk of bias was assessed independently by two reviewers using PROBAST-AI. Overall, methodological quality varied across studies, and the analysis domain was the most frequent source of concern (Table 2). Studies based on prospectively collected or real-world screening cohorts with external validation generally demonstrated low risk of bias across domains, including evaluations conducted in routine screening or clinical settings [2,28]. In contrast, several retrospective studies—particularly those conducted in tertiary referral centers, enriched case–control-like samples, or public datasets—showed unclear or high risk in the participants domain due to selection and spectrum concerns, which may limit generalizability to population-based screening [27,29,30].

Across studies, the predictors and outcome domains were largely judged as low risk because most investigations used fundus photograph-based inputs aligned with intended use, and reference standards were typically based on AREDS or Beckman criteria with expert grading and adjudication procedures [4,5,6,7,10,11,31,32]. However, the study evaluating a vision-language multimodal large language model was judged at higher risk in the predictors domain due to uncertainties regarding model version stability, reproducibility, and deployment consistency [27].

Analysis-domain concerns were common, especially in earlier retrospective studies, and were driven by factors such as limited effective sample sizes/events, reliance on internal validation, incomplete reporting of prespecified decision thresholds, and limited assessment of calibration or clinical operating points [3,5,10,32]. Although several studies reported high discrimination, these limitations reduce certainty regarding real-world clinical utility. Overall, studies with external validation and clearer reporting were interpreted as more deployment-relevant, whereas internally validated studies were considered more hypothesis-generating.

### 3.5. Overall Meta-Analysis of Deep Learning Algorithms

**Figure 2:**
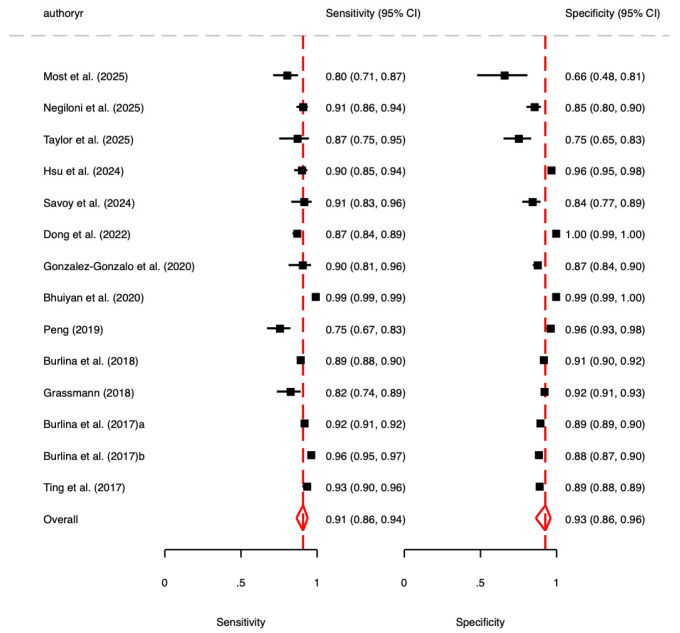
Forest plot showing the sensitivity and specificity of deep learning algorithms for detecting rAMD [3,4,5,6,7,10,11,27,28,29,30,31,32]. Red dashed vertical lines indicate the pooled (overall) sensitivity and specificity estimates.

**Figure 3:**
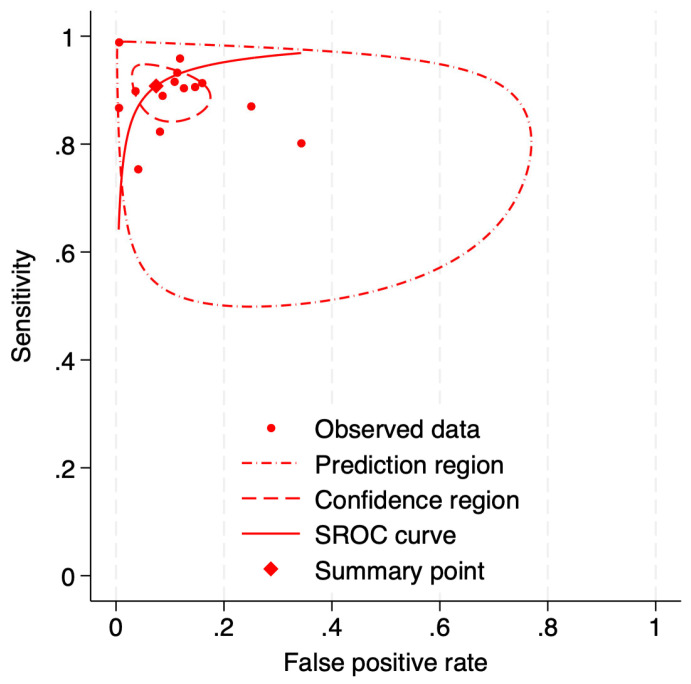
Summary receiver operating characteristic (SROC) curve visually demonstrates the diagnostic accuracy of deep learning algorithms. The dashed and dash-dotted ellipses indicate the 95% confidence and 95% prediction regions, respectively.

A total of 14 studies were included in the bivariate random-effects meta-analysis to evaluate the diagnostic performance of deep learning algorithms for detecting referable AMD. The pooled sensitivity was 0.91 (95% CI: 0.86–0.94), and the pooled specificity was 0.93 (95% CI: 0.86–0.96). Substantial heterogeneity was observed across the included studies, with I2 values of 86.75% for sensitivity and 92.94% for specificity, indicating significant variability beyond sampling error. The correlation coefficient (rho) between sensitivity and specificity was 0.38, suggesting a moderate positive correlation and the presence of a threshold effect. Individual study performance varied considerably; sensitivity estimates ranged from 0.75 (Peng et al. [32]) to 0.99 (Bhuiyan et al. [31]), while specificity estimates ranged from 0.66 (Most et al. [27]) to 1.00 (Dong et al. [4]). These results, including the corresponding forest plots, are presented in Figure 2, while the Summary Receiver Operating Characteristic (SROC) plot is depicted in Figure 3. No significant publication bias was observed for results (*p* = 0.72, Supplementary Figure S1).

### 3.6. Subgroup Analysis

Table 3 summarizes subgroup analyses conducted to explore potential sources of heterogeneity across study- and implementation-level characteristics. Overall, pooled diagnostic performance was generally consistent across prespecified covariates, and no moderator reached conventional statistical significance (all *p* > 0.05). Several non-significant patterns were noted. Studies with vendor involvement showed a trend toward higher specificity than those without (0.96 vs. 0.88; *p* = 0.07), with similar sensitivity (0.93 vs. 0.89; *p* = 0.21). Healthcare setting showed a marginal difference in sensitivity, with tertiary settings demonstrating higher sensitivity than primary-care settings (0.94 vs. 0.88; *p* = 0.08), while specificity was comparable (0.94 vs. 0.92; *p* = 0.73). Studies using public datasets had similar pooled sensitivity/specificity to those using private datasets (0.92/0.93 vs. 0.89/0.92; *p* = 0.49 and *p* = 0.81, respectively). External validation was associated with slightly higher specificity (0.94 vs. 0.91; *p* = 0.57) and similar sensitivity (0.91 vs. 0.90; *p* = 0.88). Other factors—including study design, economic status, presence of other disease targets, camera type (desktop vs. smartphone), grading criteria (AREDS vs. Beckman), risk of bias, and article type—did not materially change pooled estimates. Interpretation of some subgroup results is limited by small numbers in certain categories (e.g., smartphone imaging, Beckman criteria, low risk of bias), leading to wider uncertainty. Collectively, these findings suggest that pooled sensitivity and specificity are reasonably stable across diverse settings, while remaining heterogeneity likely reflects combined differences in case mix, reference-standard procedures, imaging conditions, and reporting practices.

### 3.7. Clinical Applicability

**Figure 4:**
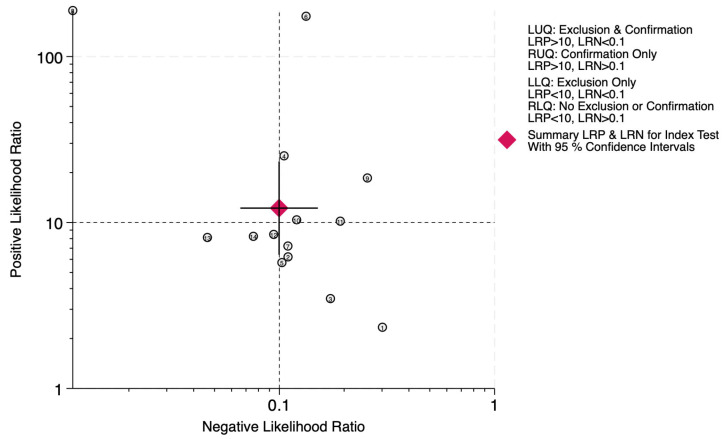
Likelihood ratio scattergram. Circled numbers indicate the study ID.

**Figure 5:**
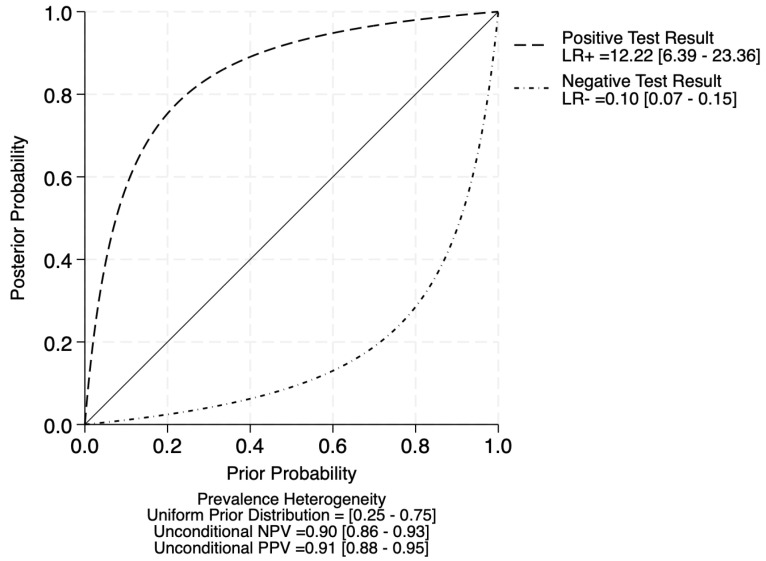
Probability modifying plot. The solid diagonal line indicates posterior probability = prior probability (LR = 1; no change in probability).

The clinical applicability of deep learning algorithms was evaluated using likelihood ratio scattergrams and probability-modifying plots (Figure 4 and Figure 5). The pooled positive likelihood ratio (PLR) was 12.22 (95% CI: 6.39–23.36), and the negative likelihood ratio (NLR) was 0.10 (95% CI: 0.07–0.15). In the likelihood ratio scattergram, the summary point was positioned near the border of the Left Upper Quadrant (LUQ)—characterized by a PLR >10 and an NLR ≈0.1—indicating strong utility for both the confirmation and exclusion of disease. These findings confirm that deep learning algorithms achieve high diagnostic accuracy and generate clinically meaningful shifts in disease probability, supporting their utility for enhancing patient triage and referral efficiency in referable AMD screening programs.

### 3.8. Contrast-Based Meta-Analysis of Deep Learning Versus Human Graders

**Figure 6:**
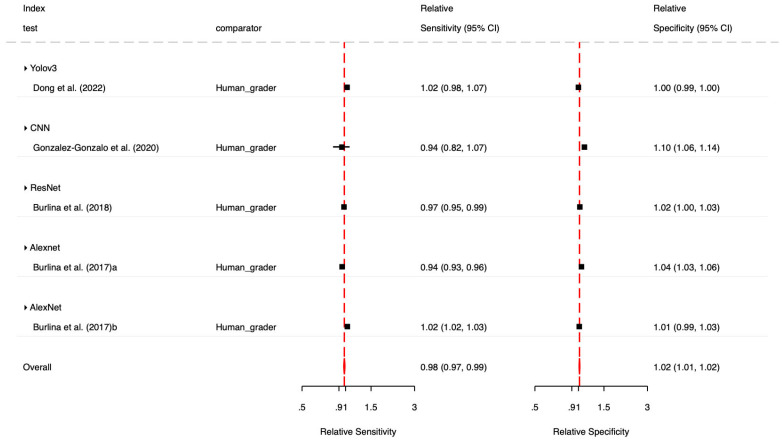
Forest plot of contrast-based meta-analysis of deep learning versus human graders [3,4,5,30,31].

The contrast-based meta-analysis, utilizing paired head-to-head data (Figure 6), revealed distinct performance trade-offs between artificial intelligence and human graders. Overall, deep learning systems demonstrated a slightly lower sensitivity compared to human experts (Pooled Relative Sensitivity: 0.98; 95% CI: 0.97–0.99), indicating that AI missed a marginally higher proportion of referable AMD cases. However, AI exhibited superior specificity (Pooled Relative Specificity: 1.02; 95% CI: 1.01–1.02), suggesting greater effectiveness than humans at reducing false-positive referrals. Significant heterogeneity was observed across different network designs (Wald test: *p* < 0.001). Among the specific architectures evaluated, AlexNet achieved the most balanced performance relative to clinicians, demonstrating the highest relative specificity (1.03) and a sensitivity nearly equivalent to human grading (0.99). In contrast, the YOLOv3 architecture matched human specificity (1.00) but showed lower relative sensitivity (0.97). The studies contributing paired AI-versus-human comparisons and the corresponding human comparator definitions are summarized in Supplementary Table S6.

## 4. Discussion

### 4.1. Principal Findings

In this systematic review and meta-analysis of 14 diagnostic accuracy studies, deep learning (DL) systems demonstrated high overall performance for detecting referable age-related macular degeneration (rAMD) from color fundus photographs, with a pooled sensitivity of 0.91 and specificity of 0.93. From a screening and triage perspective, the pooled likelihood ratios (PLR ≈ 12; NLR ≈ 0.10) indicate that a positive DL result can substantially increase the post-test probability of rAMD, while a negative result meaningfully reduces it. Collectively, these findings support the premise that DL-based grading can help address the real-world bottleneck of limited expert retinal grading capacity as imaging volumes expand, potentially strengthening referral pathways for intermediate and late AMD where timely evaluation is most valuable [1,5,6,10,18].

### 4.2. Relationship to Prior Evidence

Our findings are consistent with the broader literature showing that DL can achieve strong diagnostic accuracy in retinal imaging tasks and may reach or approach expert-level performance under certain conditions [8,9,10,18,19]. Prior systematic review evidence has also supported favorable diagnostic performance of DL for AMD detection across heterogeneous study designs and datasets [12]. The present review extends that evidence base by focusing specifically on referable AMD within a diagnostic test accuracy (DTA) framework and by incorporating newer studies and deployment-relevant settings—including real-world evaluations and smartphone-based screening approaches [11,28,29]. This matters because “high performance in development datasets” is not the same thing as “trustworthy performance in clinics,” especially when disease definitions, reference standards, image acquisition conditions, and dataset provenance vary [17,23]. In particular, repeated evaluation on publicly available research datasets may over-represent specific case mixes and labeling conventions, highlighting the importance of external validation in representative clinical populations.

### 4.3. DL Versus Human Graders: A Clinically Relevant Trade-Off

Using paired head-to-head comparisons, our contrast-based meta-analysis suggested that DL systems had slightly lower sensitivity than human graders (relative sensitivity < 1) but higher specificity (relative specificity > 1). This pattern is clinically interpretable: in many screening programs, humans may err on the side of caution, increasing sensitivity at the expense of more false positives; conversely, algorithms may be tuned (explicitly or implicitly) to reduce false positives and stabilize performance, yielding higher specificity. Similar “trade-off” themes have been noted in comparisons between automated systems and clinicians in medical AI, where study design choices, thresholds, and prevalence strongly shape apparent performance [17].

From an implementation perspective, the key point is not that “AI is better” or “humans are better,” but that threshold selection and workflow goals should drive operating points. If a program prioritizes minimizing missed rAMD (e.g., community screening in high-risk older adults), decision thresholds can be tuned toward higher sensitivity, accepting more referrals; if capacity constraints are severe, higher specificity may be preferred to reduce unnecessary referrals. These considerations are central for AMD because “referable” disease definitions typically align with intermediate or advanced stages where progression risk and management implications are highest [1,2,3,4,16].

### 4.4. Sources of Heterogeneity and Why They Matter

Despite robust pooled performance, we observed substantial heterogeneity (high I^2^ for both sensitivity and specificity), and a moderate sensitivity–specificity correlation consistent with threshold effects. This heterogeneity is not surprising in AMD DL research and likely reflects multiple interacting factors:

#### 4.4.1. Differences in the Definition of “Referable AMD”

Most studies used AREDS-based grading frameworks (simplified or multi-step variants), but operational definitions of “referable” can still differ across studies (e.g., which AREDS categories are grouped, whether late AMD is separated, and how mixed pathology is handled) [7,10,32]. Even small definitional shifts can materially affect sensitivity and specificity when pooled.

#### 4.4.2. Reference Standards and Adjudication

Human expert grading served as the reference standard in nearly all included studies, and several employed adjudication or consensus procedures to resolve inter-grader disagreement. However, grading frameworks, grader training/experience, reading-center workflows, and blinding practices varied across studies, which can affect outcome labels and, in turn, the apparent performance of deep learning models [5,10,17]. This issue is particularly salient for “borderline” presentations (e.g., subtle intermediate changes or coexisting pathology), where small differences in interpretation or adjudication rules may shift cases across the referable/non-referable threshold and contribute to between-study heterogeneity.

#### 4.4.3. Imaging Devices, Field of View, and Image Quality

Most studies relied on conventional desktop fundus cameras with 30–45° fields, but some evaluated smartphone imaging or ultrawide-field (UWF) systems [11,27,28]. Camera type can influence the image characteristics presented to DL models, including macula-centered detail, illumination uniformity, and artifact patterns, which may contribute to dataset shift between development and deployment. Smartphone-based fundus photography—while attractive for portability and access—can be more variable in acquisition quality in real-world use (e.g., inconsistent focus, glare, or uneven illumination), potentially increasing borderline-quality or ungradable images and affecting measured accuracy [11,28]. Consistent with this implementation concern, our subgroup analysis showed similar pooled sensitivity between desktop and smartphone studies, but smartphone-based studies showed lower pooled specificity with wide uncertainty; notably, this subgroup was informed by only two studies and should be interpreted cautiously [11,28].

UWF imaging provides substantially larger retinal coverage but differs from conventional fundus photography in image representation and scale; when models are developed primarily on conventional fundus images, additional validation (and potentially adaptation) may be needed to ensure robust performance on UWF images in practice [27]. Overall, these observations reinforce the importance of device-stratified external validation and transparent reporting of camera type and image-quality handling to support trustworthy clinical translation and applicability judgments [17,23].

#### 4.4.4. Handling of Ungradable Images

Real-world screening programs inevitably encounter ungradable images. Approaches vary: excluding ungradable images can inflate apparent performance, whereas classifying them as “referable” increases sensitivity but may reduce specificity, and placing them into a separate class changes the clinical interpretation entirely [6,7]. Because “ungradable” itself is a clinically actionable outcome (often requiring re-imaging or referral), consistent reporting and handling are critical for translating accuracy metrics into workflow decisions.

#### 4.4.5. Model Families and Non-Traditional Architectures

Most included systems were CNN-based, but at least one recent study evaluated a multimodal large language model approach [27]. Performance differences across architectures could reflect not just “algorithm strength” but also differences in training data, supervision signals, and how outputs are calibrated for diagnostic decisions. As these model families diversify, the field will need more standardized evaluation practices to keep comparisons honest.

#### 4.4.6. Dataset Provenance (Public vs. Private) and Population Spectrum

A further source of heterogeneity is dataset provenance and spectrum differences between publicly available research datasets and private/clinical cohorts. In subgroup analyses, studies using public datasets showed pooled performance comparable to those using private datasets (sensitivity 0.92 vs. 0.89; *p* = 0.49; specificity 0.93 vs. 0.92; *p* = 0.81). Although these differences were not statistically significant, public datasets may still differ systematically in case mix, imaging protocols, and labeling conventions, and repeated reliance on the same foundational datasets may limit generalizability to real-world screening settings. Accordingly, pooled estimates should be interpreted in light of potential dataset-domain effects, and future work should emphasize representative, prospectively collected cohorts and device-stratified external validation.

#### 4.4.7. Methodological Quality and Risk of Bias

Variation in methodological quality can also influence observed performance. When stratified by PROBAST-AI overall risk of bias, studies rated as unclear risk showed higher pooled sensitivity and specificity (0.93 and 0.95, respectively) than those rated as high risk (0.88 and 0.89), although differences were not statistically significant (*p* = 0.37 for sensitivity; *p* = 0.58 for specificity). These patterns should be interpreted cautiously, as subgroup sizes were small (particularly the low-risk subgroup), and risk-of-bias categories may correlate with other study characteristics (e.g., dataset source, validation design, and reporting quality). Nevertheless, the direction of effect is clinically plausible: selection/spectrum bias, unclear threshold pre-specification, and incomplete handling of non-independence can inflate or distort apparent model performance. This finding reinforces the need for standardized reporting and robust external validation to support deployment-relevant estimates.

### 4.5. Implications for Screening, Triage, and Service Delivery

High pooled specificity suggests DL could reduce false-positive referrals, which is particularly valuable in systems with limited retina specialist capacity. At the same time, the pooled NLR indicates DL can meaningfully reduce post-test probability when negative, supporting its role as a triage tool in large-scale screening. These advantages align with the broader movement toward AI-supported retinal disease referral systems [8,18,19].

Several included studies also reflect the “real-world direction of travel”: multi-disease screening pipelines (e.g., DR + AMD) and portable imaging solutions may help reach underserved populations [6,11,28,30]. From a public health standpoint, such deployment could complement existing screening infrastructure (especially diabetic eye screening programs) and potentially support earlier identification of at-risk AMD, although the clinical value ultimately depends on downstream pathways and access to treatment [2,3,4,16].

Importantly, fundus photography alone may not optimally detect all exudative changes compared with OCT-based evaluation; thus, DL fundus screening is best conceptualized as a referral-enabling front end, not a replacement for specialist assessment or multimodal clinical imaging [7,8,20]. Emerging workflows (including home monitoring technologies) underscore that AMD management increasingly spans beyond single-visit fundus assessment, creating opportunities for future AI systems that combine modalities and longitudinal risk prediction [14,20].

### 4.6. Methodological Strengths and Limitations

This review followed PRISMA-DTA guidance and applied an AI-aware risk-of-bias framework using PROBAST-AI principles [21,23]. We also used bivariate random-effects meta-analysis and explored heterogeneity via subgroup analyses, which is appropriate for DTA synthesis. Nevertheless, several limitations should be considered: Modality restriction: We focused on color fundus photography, excluding OCT and angiography. Although OCT is referenced to contextualize clinical workflows and confirmatory evaluation, our meta-analysis is restricted to fundus photograph-based DL systems, and the findings should not be extrapolated to AI models that use OCT or other imaging modalities as inputs. This improves comparability but limits inference to fundus-only pathways, which may be less sensitive for certain neovascular features [7,20]. Study design mix: Many included studies were retrospective and/or used public datasets. Retrospective evaluation can overestimate real-world performance when case selection and image quality differ from screening practice [17,23]. Variable reporting: Not all studies report confusion matrices, operating thresholds, calibration, or subgroup performance (e.g., by age, media opacity, or comorbidity), limiting deeper synthesis and fairness assessment [17,23]. Non-independence issues: Some studies analyze both eyes or multiple images per patient without fully accounting for clustering, which can bias precision estimates and inflate apparent confidence [23]. Head-to-head evidence: The AI-versus-human contrast analysis relies on paired comparisons; the number of such datasets is typically smaller than the total number of AI-only validation studies, which can constrain conclusions about comparative performance.

### 4.7. Future Directions

Future research should prioritize: (i) prospective, multi-center external validation in representative screening populations; (ii) standardized and clinically meaningful definitions of “referable AMD” aligned with treatment and monitoring pathways [2,3,4,16]; (iii) transparent reporting of thresholds, calibration, and handling of ungradable images [6,7]; and (iv) evaluation of clinical impact, including referral burden, time-to-treatment, and cost-effectiveness rather than accuracy metrics alone [17,18]. As the evidence base expands, future meta-analyses should also apply multivariable meta-regression to jointly assess key moderators (e.g., case mix, reference standards, device type, validation design, and vendor involvement), enabling more robust explanation of heterogeneity than univariable analyses alone. Finally, integrating cross-sectional detection with longitudinal risk prediction (e.g., progression to neovascular AMD) may deliver greater clinical value than classification alone and better support personalized surveillance strategies [14].

## 5. Conclusions

Deep learning algorithms demonstrate high diagnostic accuracy for detecting referable age-related macular degeneration on color fundus photographs, with pooled sensitivity and specificity exceeding 0.90 and clinically meaningful likelihood ratios. Beyond accuracy, DL offers practical benefits for service delivery, including rapid and scalable image triage, more standardized grading that may reduce expert workload and inter-reader variability, and configurable operating thresholds that can improve referral efficiency (e.g., reducing false-positive referrals when specialist capacity is constrained). DL systems may also facilitate teleophthalmology and portable screening models and integrate into multi-disease retinal screening pipelines. However, substantial heterogeneity and recurring methodological limitations highlight the need for standardized disease definitions, transparent reporting (including thresholds and handling of ungradable images), and prospective, device-stratified external validation before widespread real-world implementation.

## Figures and Tables

**Table 1:** Basic characteristics of included studies.

Study (Ref)	Design	Study Period	Source/Setting	Country	Population	Age (Reported)	Female %	Sample Size (Patients/Images)	Other Targets	Income Category	AMD Target
Most et al., 2025 [27]	Retrospective	Apr 2023–Jun 2024 (collection); Dec 2024–Feb 2025 (testing)	UCSD Shiley Eye Institute; tertiary	United States	Adults	Mean 81.1	69.7	76/136	None	High income	Referable AMD
Negiloni et al., 2025 [28]	Prospective	Nov 2022–Nov 2023	Aravind Eye Hospital (Chennai); tertiary	India	Adults ≥40	Mean 61.8 ± 9.9	56.0	492/984	None	Lower-middle income	Referable AMD
Taylor et al., 2025 [29]	Retrospective	Jan 2020–Jun 2022	Rural ophthalmology clinics; primary care	Australia	High-risk rural adults (known DR or AMD)	Mean 64.0 ± 12.8	51.0	82/150	Diabetic retinopathy	High income	Referable AMD
Hsu et al., 2024 [2]	Retrospective	Dev Aug 2010–Jun 2019; Val Jul 2019–Apr 2021	NTUH; tertiary	Taiwan	Adults ≥50	NR	NR	NR/7738	None	High income	Referable AMD
Savoy et al., 2024 [11]	Retrospective	Mar 2013–Oct 2020; Jan 2022–Mar 2022	AREDS + South Asian target-device evaluation; mixed settings	United States; India	Adults (AREDS + South Asian cohorts)	Mean 51.9	44.3 †	AREDS: 909/108,251; Target: 238/1108	None	Mixed (High + Lower-middle)	Referable AMD
Dong et al., 2022 [4]	Prospective	Dev Jun 2018–Jun 2020; Val Nov 2020–Feb 2021	iKang health check-up centers; primary care	China	General screening population	Median 42 (range 8–87)	55.3	Dev: 63,400/120,002; Val: 110,784/208,758	Multiple (e.g., DR and glaucoma)	Upper-middle income	Referable AMD
González-Gonzalo et al., 2020 [30]	Retrospective	Images acquired Aug 2011–Oct 2016	Routine clinical practice + public datasets	Europe; United States	Routine retinal imaging patients	NR	NR	DR-AMD: 288/600; Messidor: NR/1200; AREDS: NR/133,821	Diabetic retinopathy	High income (predominant)	Referable AMD
Bhuiyan et al., 2020 [31]	Retrospective	AREDS long-term follow-up (~12 years)	AREDS research cohorts	France	Adults (AREDS participants)	Mean 69.4	55.7	4753/16,875	None	High income	Referable AMD
Peng et al., 2019 [32]	Retrospective	AREDS follow-up (~12 years)	AREDS dataset	United States	Adults (AREDS participants)	NR	NR	4549/59,302	None	High income	Referable AMD
Burlina et al., 2018 [10]	Retrospective	Nov 1992–Nov 2005	AREDS study period	United States	Adults (AREDS participants)	NR	NR	4613/67,401	None	High income	Referable AMD
Grassmann et al., 2018 [7]	Retrospective	AREDS follow-up (~12 years)	AREDS + KORA cohorts	Germany	Adults (AREDS; KORA analyses)	NR	NR	AREDS: 3654/120,656; KORA: 5555/NR	None	High income	Referable AMD
Burlina et al., 2017a [5]	Retrospective	AREDS follow-up (~12 years)	AREDS dataset	United States	Adults (AREDS participants)	NR	NR	4613/133,821	None	High income	Referable AMD
Burlina et al., 2017b [3]	Retrospective	AREDS follow-up (~12 years)	AREDS dataset	United States	Adults (AREDS participants)	>50 (reported)	NR	NR/5664	None	High income	Referable AMD
Ting et al., 2017 [6]	Retrospective	Train 2010–2013; Val 2014–2017	National DR screening + multi-country validation	Singapore + multi-country	Adults with diabetes	Mean 60.2	45.4	14,880/35,948	Diabetic retinopathy (and related eye diseases)	Mixed (multi-country)	Referable AMD

Abbreviation: AMD: Age-Related Macular Degeneration; AREDS: Age-Related Eye Disease Study; DR: Diabetic Retinopathy; KORA: Cooperative Health Research in the Region of Augsburg; NR: Not Reported; NTUH: National Taiwan University Hospital; UCSD: University of California, San Diego; USA: United States of America.

**Table 2:** PROBAST-AI assessment of risk of bias and applicability of included studies.

Study (Year)	Participants	Predictors (AI Model)	Outcome (Reference Standard)	Analysis	Overall ROB
Most et al. (2025) [27]	High	High	Low	High	High
Negiloni et al. (2025) [28]	Low	Low	Low	Low	Low
Taylor et al. (2025) [29]	Unclear	Low	Low	Unclear	Unclear
Hsu et al. (2024) [2]	Low	Low	Low	Low	Low
Savoy et al. (2024) [11]	Unclear	Low	Low	Unclear	Unclear
Dong et al. (2022) [4]	Low	Low	Low	Unclear	Unclear
Gonzalez-Gonzalo et al. (2020) [30]	Unclear	Low	Low	Unclear	Unclear
Bhuiyan et al. (2020) [31]	Low	Low	Low	Unclear	Unclear
Peng (2019) [32]	Low	Low	Low	High	High
Burlina et al. (2018) [10]	Low	Low	Low	High	High
Grassmann (2018) [7]	Unclear	Low	Low	High	High
Burlina et al. (2017) a [5]	Low	Low	Low	High	High
Burlina et al. (2017) b [3]	Low	Low	Low	High	High
Ting et al. (2017) [6]	Low	Low	Low	Unclear	Unclear

**Table 3:** Analysis Based on Various Sub-groups.

Moderator	Category	N	Deep Learning Algorithms			
			Sensitivity	*p*-Value	Specificity	*p*-Value
Study design	Retrospective	10	0.91 (0.87–0.94)	0.67	0.91 (0.84–0.95)	0.21
	Prospective	4	0.89 (0.72–0.96)		0.97 (0.86–0.99)	
Economic status	High	11	0.91 (0.86–0.94)	0.74	0.92 (0.84–0.96)	0.53
	Low-Middle	3	0.90 (0.77–0.96)		0.95 (0.81–0.99)	
Healthcare setting	Primary	9	0.88 (0.82–0.92)	0.08	0.92 (0.83–0.96)	0.73
	Tertiary	5	0.94 (0.89–0.97)		0.94 (0.82–0.98)	
Dataset	Public dataset	5	0.92 (0.87–0.95)	0.49	0.93 (0.85–0.97)	0.81
	Private dataset	8	0.89 (0.80–0.94)		0.92 (0.78–0.97)	
External validation	Yes	9	0.91 (0.85–0.95)	0.88	0.94 (0.86–0.97)	0.57
	No	5	0.90 (0.82–0.95)		0.91 (0.76–0.97)	
Other target	Yes	4	0.90 (0.79–0.95)	0.74	0.93 (0.79–0.98)	0.87
	No	10	0.91 (0.86–0.94)		0.92 (0.84–0.96)	
Camera type	Desktop	12	0.91 (0.86–0.94)	0.92	0.93 (0.87–0.97)	0.35
	Smartphone	2	0.91 (0.76–0.97)		0.85 (0.49–0.97)	
Criteria	AREDS	10	0.91 (0.87–0.94)	0.41	0.92 (0.84–0.96)	0.39
	Beckman	2	0.86 (0.67–0.95)		0.96 (0.81–0.99)	
Vendor involved	No	8	0.89 (0.82–0.93)	0.21	0.88 (0.77–0.94)	0.07
	Yes	6	0.93 (0.88–0.96)		0.96 (0.91–0.98)	
Risk of bias	High	6	0.88 (0.80–0.93)	0.37	0.89 (0.76–0.96)	0.58
	Unclear	6	0.93 (0.88–0.96)		0.95 (0.87–0.98)	
	Low	2	0.90 (0.77–0.96)		0.93 (0.69–0.99)	
Article type	Original	14	0.91 (0.87–0.94)	0.51	0.93 (0.87–0.96)	0.27
	Supplement	2	0.87 (0.68–0.95)		0.90 (0.61–0.98)	

Abbreviation: AREDS: Age-Related Eye Disease Study.

## Data Availability

Data available on request due to privacy and ethical restrictions.

## Supplementary Materials

The following supporting information can be downloaded at: https://www.mdpi.com/article/10.3390/diagnostics16040633/s1, Table S1: PRISMA-DTA Abstract Checklist; Table S2: PRISMA-DTA Checklist; Table S3: Keywords and search results in different database; Table S4: Study-by-study operational definitions of referable AMD, handling of ungradable images, and unit of analysis (per-image/per-eye/per-patient) across included studies; Table S5: Comparative Overview of AI Deep Learning Algorithms in Diagnostic Studies; Table S6: Comparative Overview of AI Deep Learning Algorithms in Diagnostic Studies; Figure S1: Deek’s test plot.

## Abbreviations

The following abbreviations are used in this manuscript:

AMD	Age-related macular degeneration
rAMD	Referable age-related macular degeneration
DL	Deep learning
AI	Artificial intelligence
CNN	Convolutional neural network
OCT	Optical coherence tomography
AREDS	Age-Related Eye Disease Study
DTA	Diagnostic test accuracy
PRISMA-DTA	Preferred Reporting Items for Systematic Reviews and Meta-Analyses of Diagnostic Test Accuracy
PROBAST	Prediction model Risk Of Bias ASsessment Tool
PROBAST-AI	PROBAST adapted for artificial intelligence models
TP	True positive
FP	False positive
TN	True negative
FN	False negative
CI	Confidence interval
SROC	Summary receiver operating characteristic
PLR	Positive likelihood ratio
NLR	Negative likelihood ratio
UWF	Ultra-wide field
ViT	Vision Transformer
