# Supplementary material for: Performance and Clinical Utility of Deep Learning for Detecting Referable Age-Related Macular Degeneration on Fundus Photographs: A Systematic Review and Meta-Analysis
Source: Diagnostics (Basel). 2026 Feb 22;16(4):633. doi: 10.3390/diagnostics16040633 (PMC12939426; doi:10.3390/diagnostics16040633)
Supplement: Supplementary file 1 [file diagnostics-16-00633-s001.zip › diagnostics-4101396-supplementary.pdf]

Table S1. PRISMA-DTA Abstract Checklist.

Section/Topic	Number	PRISMA-DTA for Abstracts Checklist Item	Reported on Page #
		TITLE and PURPOSE	
Title	1	Identify the report as a systematic review (+/- meta-analysis) of diagnostic test accuracy (DTA) studies.	Title page
Objectives	2	Indicate the research question, including components such as participants, index test, and target conditions.	1
		METHODS	
Eligibility criteria	3	Include study characteristics used as criteria for eligibility.	1
Information sources	4	List the key databases searched and the search dates.	1
Risk of bias & applica-bility	5	Indicate the methods of assessing risk of bias and applicability.	1
Synthesis of results	A1		1
		RESULTS	
Included studies	6	Indicate the number and type of included studies and the participants and rele-vant characteristics of the studies (including the reference standard).	1
Synthesis of results	7	Include the results for the analysis of diagnostic accuracy, preferably indicating the number of studies and participants. Describe test accuracy including varia-bility; if meta-analysis was done, include summary results and confidence inter-vals.	1
		DISCUSSION	
Strengths and limita-tions	9	Provide a brief summary of the strengths and limitations of the evidence	1
Interpretation.	10	Provide a general interpretation of the results and the important implications.	1
		OTHER	
Funding	11	Indicate the primary source of funding for the review	NA
Registration	12	Provide the registration number and the registry name	NA

Adapted From: McInnes MDF, Moher D, et al. The PRISMA-DTA Group (2018). Preferred Reporting Items for a Systematic Review and Meta-analysis of Diagnostic Test Accuracy Studies: The PRISMA-D

Table S2. PRISMA-DTA Checklist.

Section/Topic	Number	PRISMA-DTA for Abstracts Checklist Item	Reported on Page #
		TITLE and PURPOSE	
Title	1	Identify the report as a systematic review (+/- meta-analysis) of diagnostic test accuracy (DTA) studies.	Title page
Abstract	2	Abstract: See PRISMA-DTA for abstracts.	1
		INTRODUCTION	
Rationale	3	Describe the rationale for the review in the context of what is already known.	2

Clinical role of index test	D1	State the scientific and clinical background, including the intended use and clinical role of the index test, and if applicable, the rationale for minimally acceptable test accuracy (or minimum difference in accuracy for comparative design).	2
Objectives	4	Provide an explicit statement of question(s) being addressed in terms of participants, index test(s), and target condition(s).	2
		METHODS	
Protocol and registration	5	Indicate if a review protocol exists, if and where it can be accessed (e.g., Web address), and, if available, provide registration information including registration number.	3
Eligibility criteria	6	Specify study characteristics (participants, setting, index test(s), reference standard(s), target condition(s), and study design) and report characteristics (e.g., years considered, language, publication status) used as criteria for eligibility, giving rationale.	3
Information sources	7	Describe all information sources (e.g., databases with dates of coverage, contact with study authors to identify additional studies) in the search and date last searched.	3
Search	8	Present full search strategies for all electronic databases and other sources searched, including any limits used, such that they could be repeated.	3
Study selection	9	State the process for selecting studies (i.e., screening, eligibility, included in systematic review, and, if applicable, included in the meta-analysis).	3
Data collection process	10	Describe method of data extraction from reports (e.g., piloted forms, independently, in duplicate) and any processes for obtaining and confirming data from investigators.	3-4
Definitions for data extraction	11	Provide definitions used in data extraction and classifications of target condition(s), index test(s), reference standard(s) and other characteristics (e.g. study design, clinical setting).	3-4
Risk of bias and applicability	12	Describe methods used for assessing risk of bias in individual studies and concerns regarding the applicability to the review question.	4
Diagnostic accuracy measures	13	State the principal diagnostic accuracy measure(s) reported (e.g. sensitivity, specificity) and state the unit of assessment (e.g. per-patient, per-lesion).	4
Synthesis of results	14	Describe methods of handling data, combining results of studies and describing variability between studies. This could include, but is not limited to: a) handling of multiple definitions of target condition. b) handling of multiple thresholds of test positivity, c) handling multiple index test readers, d) handling of indeterminate test results, e) grouping and comparing tests, f) handling of different reference standards	4
Meta-analysis	D2	Report the statistical methods used for meta-analyses, if performed.	4
Additional analyses	16	Describe methods of additional analyses (e.g., sensitivity or subgroup analyses, meta-regression), if done, indicating which were pre-specified.	4

RESULTS			
Study selection	17	Provide numbers of studies screened, assessed for eligibility, included in the review (and included in meta-analysis, if applicable) with reasons for exclusions at each stage, ideally with a flow diagram	5
Study characteristics	18	For each included study provide citations and present key characteristics including: a) participant characteristics (presentation, prior testing), b) clinical setting, c) study design, d) target condition definition, e) index test, f) reference standard, g) sample size, h) funding sources	5
Risk of bias and applicability	19	Present evaluation of risk of bias and concerns regarding applicability for each study.	6
Results of individual studies	20	For each analysis in each study (e.g. unique combination of index test, reference standard, and positivity threshold) report 2x2 data (TP, FP, FN, TN) with estimates of diagnostic accuracy and confidence intervals, ideally with a forest or receiver operator characteristic (ROC) plot.	N/A (individual 2×2 tables not shown)
Synthesis of results	21	Describe test accuracy, including variability; if meta-analysis was done, include results and confidence intervals	7-9
Additional analysis	23	Give results of additional analyses, if done (e.g., sensitivity or subgroup analyses, meta-regression; analysis of index test: failure rates, proportion of inconclusive results, adverse events).	7-9
DISCUSSION			
Summary of evidence	24	Summarize the main findings including the strength of evidence	8
Limitations	25	Discuss limitations from included studies (e.g. risk of bias and concerns regarding applicability) and from the review process (e.g. incomplete retrieval of identified research).	8-10
Conclusions	26	Provide a general interpretation of the results in the context of other evidence. Discuss implications for future research and clinical practice (e.g. the intended use and clinical role of the index test)	11
OTHER			
Funding	27	For the systematic review, describe the sources of funding and other support and the role of the funders	NA

Adapted From: McInnes MDF, Moher D, et al. The PRISMA-DTA Group (2018). Preferred Reporting Items for a Systematic Review and Meta-analysis of Diagnostic Test Accuracy Studies: The PRISMA-DTA Statement. JAMA. 2018 Jan 23;319(4):388-396. doi: 10.1001/jama.2017.19163.

Table S3. Keywords and search results in different database

Database	Keyword	Date	Results
PubMed	("Deep Learning"[MeSH] OR "Neural Networks, Computer"[MeSH] OR "Artificial	2025/12/17	717

	Intelligence"[MeSH] OR deep learning[tiab] OR deep neural network*[tiab] OR convolutional neural network*[tiab] OR CNN[tiab] OR DCNN[tiab] OR U-Net[tiab] OR ResNet[tiab] OR GAN[tiab] OR transformer[tiab] OR vision transformer[tiab] OR ViT[tiab] OR Swin Transformer[tiab] OR attention-based[tiab] OR self-attention[tiab]) AND ("Macular Degeneration"[MeSH] OR "Geographic Atrophy"[MeSH] OR Drusen[MeSH] OR "Choroidal Neovascularization"[MeSH] OR age-related macular degeneration[tiab] OR age related macular degeneration[tiab] OR AMD[tiab] OR macular degeneration[tiab] OR geographic atrophy[tiab] OR wet AMD[tiab] OR dry AMD[tiab] OR nAMD[tiab] OR drusen[tiab] OR choroidal neovascularization[tiab] OR CNV[tiab] OR pigment epithelial detachment[tiab] OR PED[tiab]) AND ("Fundus Photography"[MeSH] OR "Tomography, Optical Coherence"[MeSH] OR fundus[tiab] OR fundus photograph*[tiab] OR retinal image*[tiab] OR color fundus[tiab] OR optical coherence tomography[tiab] OR OCT[tiab])		
Embase	('deep learning'/exp OR 'artificial neural network'/exp OR 'deep learning':ti,ab OR 'deep neural network*':ti,ab OR 'convolutional neural network*':ti,ab OR 'cnn':ti,ab OR 'dcnn':ti,ab OR 'u-net':ti,ab OR 'resnet':ti,ab OR 'gan':ti,ab OR 'generative adversarial network':ti,ab OR 'transformer':ti,ab OR 'vision transformer':ti,ab OR 'vit':ti,ab OR 'swin transformer':ti,ab OR 'attention-based':ti,ab OR 'self-attention':ti,ab OR 'transfer learning':ti,ab OR 'ensemble learning':ti,ab) AND ('retina macula age related degeneration'/exp OR 'retina macula degeneration'/exp OR 'geographic atrophy'/exp OR 'drusen'/exp OR 'subretinal neovascularization'/exp OR 'age related macular degeneration':ti,ab OR 'amd':ti,ab OR 'macular degeneration':ti,ab OR 'geographic atrophy':ti,ab OR 'wet amd':ti,ab OR 'dry amd':ti,ab OR 'namd':ti,ab OR 'drusen':ti,ab OR 'choroidal neovascularization':ti,ab OR 'cnv':ti,ab OR 'pigment epithelial detachment':ti,ab OR 'ped':ti,ab) AND ('fundus photography'/exp OR 'optical coherence tomography'/exp OR	2025/12/17	784

	'fluorescein angiography'/exp OR 'fundus autofluorescence'/exp OR 'fundus':ti,ab OR 'fundus photograph*':ti,ab OR 'retinal image*':ti,ab OR 'color fundus':ti,ab OR 'optical coherence tomography':ti,ab OR 'oct':ti,ab OR 'octa':ti,ab OR 'oct-a':ti,ab OR 'angiography':ti,ab OR 'autofluorescence':ti,ab OR 'faf':ti,ab)		
Web of science	("deep learning" OR "deep neural network*" OR "convolutional neural network*" OR CNN OR DCNN OR "U-Net" OR ResNet OR GAN OR transformer OR "vision transformer" OR ViT OR "Swin Transformer" OR "attention-based" OR "self-attention") ("age-related macular degeneration" OR "age related macular degeneration" OR AMD OR "macular degeneration" OR "geographic atrophy" OR drusen OR "choroidal neovascularization" OR CNV OR "pigment epithelial detachment" OR PED) (fundus OR "fundus photograph*" OR "retinal image*" OR "color fundus" OR "optical coherence tomography" OR OCT)	2025/12/17	1097
IEEE	("deep learning" OR "deep neural network" OR "convolutional neural network" OR CNN OR DCNN OR "U-Net" OR ResNet OR GAN OR transformer OR "vision transformer" OR ViT OR "Swin Transformer" OR "attention-based" OR "self-attention") AND ("age-related macular degeneration" OR AMD OR "macular degeneration" OR "geographic atrophy" OR drusen OR "choroidal neovascularization") AND (fundus OR "fundus photograph" OR "retinal image" OR "optical coherence tomography" OR OCT)	2025/12/17	218

Table S4: Study-by-study operational definitions of referable AMD, handling of ungradable images, and unit of analysis (per-image/per-eye/per-patient) across included studies.

Author	Referable AMD Definition	Ungradable Handling	Unit of Analysis
Most et al. 2025 [27]	Intermediate + Late AMD (Wet/GA)	Excluded	Per-Eye
Negiloni et al. 2025 [28]	Intermediate + Late AMD	Flagged for Retake / Manual Review	Per-Patient
Taylor et al. 2025 [29]	Referable Maculopathy (Any AMD requiring specialist)	Excluded	Per-Patient
Hsu et al. 2024 [2]	Intermediate + Late AMD	Excluded	Per-Image
Savoy et al. 2024 [11]	AREDS Cat 3 & 4 (Intermediate + Late)	Excluded	Per-Image
Dong et al. 2022 [4]	Intermediate + Late AMD (Beckman Class.)	Excluded from calc (reported separately)	Per-Eye
González-Gonzalo 2020 [30]	Referable AMD (Soft drusen or worse)	Excluded	Per-Image
Bhuiyan et al. 2020 [31]	AREDS Cat 3 & 4	Excluded	Per-Image
Peng et al. 2019 [32]	AREDS Cat 3 & 4	Excluded	Per-Image
Burlina et al. 2018 [10]	AREDS Cat 3 & 4	Excluded	Per-Image
Grassmann et al. 2018 [7]	AREDS Cat 3 & 4	Excluded	Per-Image
Burlina et al. 2017a [5]	AREDS Cat 3 & 4	Excluded	Per-Image
Burlina et al. 2017b [33]	AREDS Cat 3 & 4	Excluded	Per-Image
Ting et al. 2017 [6]	Intermediate + Late AMD	Excluded (Quality filter applied)	Per-Image

Abbreviations: AMD = Age-related Macular Degeneration; AREDS = Age-Related Eye Disease Study; Cat = Category; GA = Geographic Atrophy; Wet AMD = Neovascular AMD; Beckman Class. = Beckman Initiative for Macular Research Classification.

Table S5: Comparative Overview of AI Deep Learning Algorithms in Diagnostic Studies

Author	Reference Standard	Image	Disagreement	Criteria used	Camera Type	Num of Field	Field of vision	Images used and Camera Brand	Ungradable	Training dataset	Training size	Validation method	Vendor involved	Certification	DL algorithms	Architecture
Most et al. (2025)[27]	Human expert grading	Ultrawide field (UWF) Fundus Camera	Consensus grading by two junior retinal specialists + one senior adjudicator	AREDS Classification	Desktop	1	200°	Optos P200DTx	excluded	NR	NR	External validation	No	No	Claude	Transformer
Negiloni et al. (2025) [28]	Human expert grading	Colour fundus photographs	Adjudicated by a senior retina specialist	AREDS simplified criteria (Fundus); Predefined criteria (Combined)	Smartphone	1	42°	Remidio Fundus on Phone (FOP) NM-10	excluded	USA (AREDS) and India	109359	External validation	Yes	Yes	Convolutional Neural Network (CNN) (Medios AI-AMD)	EfficientNet V.2
Taylor et al. (2025) [29]	Human expert grading	Color fundus photographs	Arbitrated by a third ophthalmologist	Beckman classification (Intermediate AMD or worse = Referable)	Desktop	1	45°	Topcon Maestro (Optical Coherence Tomography device with fundus camera)	excluded	NR	NR	External validation	No	Yes	RetCAD v1.3.0	CNN
Hsu et al. (2024) [2]	Human expert grading	Color fundus photography	Adjudicated by two senior retina specialists	AREDS 4-step severity scale	Desktop	1	45°	Canon CR-2	excluded	Taiwan	6123	External validation	Yes	Yes	VeriSee™ AMD	DenseNet
Savoy et al. (2024) [11]	Human expert grading	Colour fundus photographs	Consensus grading (Target device dataset)	AREDS grading definitions	Smartphone	1	NR (AREDS standard, ~30°)	Remidio FOP NM10 (Target Device)	excluded	USA (AREDS) and India	109359	Internal validation	Yes	Yes	Transfer Learning (CNN)	EfficientNet V.2
Dong et al. (2022) [4]	Human expert grading	Color fundus photographs	Arbitration by a third specialist	Beckman classification (Intermediate AMD or worse = Referable)	Desktop	1	45°	Canon CR6-45NM (in external validation sets); various non-mydriatic cameras in screening centers	excluded	China	100,000	External validation	Yes	No	Multitask Convolutional Neural Network (RAIDS)	Yolov3
Gonzalez-Gonzalo et al. (2020)[30]	Human expert grading	Colour fundus photographs	Single senior ophthalmologist for DR-AMD; public expert consensus for Messidor; AREDS reading-centre grades	AREDS severity scale	Desktop	1	45° (DR-AMD, Messidor); digitized analog images in AREDS	Canon CR-2PlusAF (DR-AMD); Topcon TRC-NW6 (Messidor); various digitized cameras (AREDS)	excluded	NR	NR	External validation	Yes	CE-certified (RetCAD v1.3.0)	RetCAD v1.3.0	CNN
Bhuiyan et al. (2020)[31]	Human expert grading	Color fundus photographs	Central AREDS reading-center adjudication	AREDS grading definitions	Desktop	1	~30°	Digitized film fundus photographs (AREDS; brand NR)	excluded	United States	93380	External validation	Yes	No	Deep learning + machine learning pipeline	Ensemble of CNNs: Inception-V3, Inception-ResNet-V2,

																Xception, NASNet
Peng (2019)[32]	Human expert grading	Colour fundus photographs	Compared against 88 Retinal Specialists and Reading Center grades	AREDS Simplified Severity Scale	Desktop	1	30°	Digitized film photographs / Brand NR	NR	United States	58402	Internal validation	No	No	DeepSeeNet	Inception-v3
Burlina et al. (2018)[10]	Human expert grading	Human expert grading	Human expert grading	AREDS 4-step severity scale	Desktop	1	30° (AREDS) and 45° (KORA)	NR	NR	United States	59313	Internal validation	No	No	Deep Learning (DCNN)	ResNet
Grassmann (2018)[7]	Human expert grading	Colour fundus photographs	Compared against physician grading and AREDS gold standard	AREDS 9-step severity scale	Desktop	1	30° (AREDS) and 45° (KORA)	Zeiss (AREDS); Topcon (KORA)	Included as a specific class	United States	86770	External validation	No	No	Ensemble of 6 CNNs	AlexNet, GoogLeNet, VGG, Inception-V3, ResNet, Inception-ResNet-V2
Burlina et al. (2017) a [5]	Expert human grading	Colour fundus photographs	Central reading-center adjudication	AREDS grading protocol	Desktop	1	NR (AREDS standard, ~30°)	Digitized color fundus photograph	excluded	United States	130000	Internal validation	No	No	DCNN-A	Alexnet
Burlina et al. (2017) b [33]	Human expert grading	Colour fundus photographs	Compared against physician grading and AREDS "gold standard"	AREDS grading protocol	Desktop	1	30°	Digitized analog photographs	NR	United States	5097	Internal validation	No	No	OverFeat	Alexnet
Ting et al. (2017)[6]	Human expert grading	Color fundus photographs	Senior graders with retinal specialist adjudication	AREDS grading system	Desktop	2	~45°	Topcon, Canon, Carl Zeiss, FundusVue (varies by cohort)	consider referable	Singapore + additional cohorts	72610	External validation	No	CE-certified (RetCAD v1.3.0)	SELENA+	VGGNet

Table S6: Comparative Overview of AI Deep Learning Algorithms in Diagnostic Studies

Author	Paired-comparison evidence reported?	Human comparator (definition in study)	Human comparator unit	Notes on reference standard / comparison
Hsu et al. [2]	Yes	Ophthalmologists (human performance reported alongside AI)	Per-image	Human grading reported as comparator on the test set (AREDS-based grading).
Ting et al. [6]	Yes	Trained professional graders (screening graders); retinal specialists used as reference standard	Per-eye	DL system compared against human graders; reference standard based on retinal specialist grading/adjudication.
González-Gonzalo et al. [30]	Yes	Multiple human observers (observer performance reported for comparison with DL)	Per-image	Observer study design; human performance computed on the same dataset as AI.
Burlina et al. 2017 [5]	Yes	Trained retinal specialist / expert grader (explicit human–machine comparison reported)	Per-image	Includes an explicit "human vs DL" comparison on a graded subset; ensure no overlap is double-counted if another AREDS-based Burlina paper is also included.
Burlina et al. 2017 (Comput Biol Med) [33]	Yes	Physician grading (human performance reported vs DL across classification tasks)	Per-image (AREDS CFP)	Explicit "humans vs deep learning" comparison; likely AREDS-based and may overlap with other Burlina/AREDS evaluations—avoid double-counting in primary paired meta-analysis (document choice if only one is included).

diagnostics

MDPI

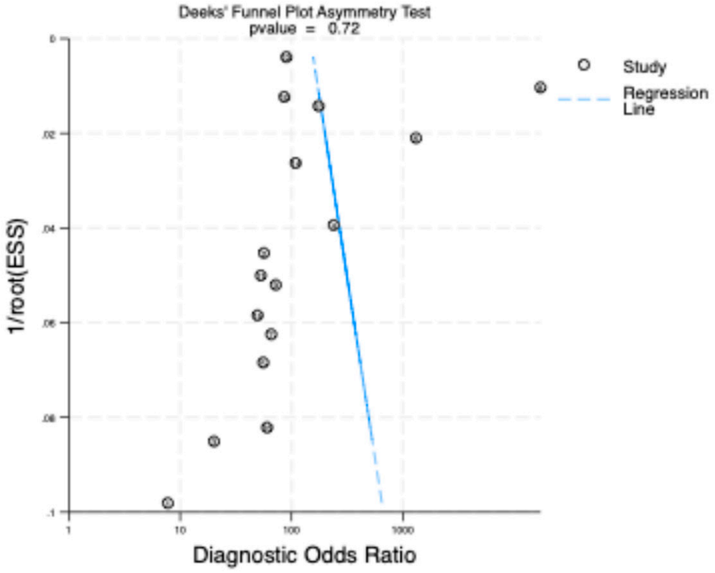

Figure S1. Deek's test plot.

*ESS: effective sample size
